# Understanding healthy eating/nutrition interventions for people living with dementia: A systematic review

**DOI:** 10.1002/alz70858_105695

**Published:** 2025-12-26

**Authors:** Laura E. Middleton, Vanessa Vucea‐Tirabassi, Liu Grace, Christine Aiken, Jennifer Bethell, Heather A. Cooke, Heather Keller, Teresa Liu‐Ambrose, Myrna Norman, Megan E O'Connell, Jackie E Stapleton, Ingrid Waldron, Sarah A. Wu, Marie‐Lee Yous, Carrie A McAiney

**Affiliations:** ^1^ Schlegel‐UW Research Institute for Aging, Waterloo, ON, Canada; ^2^ University of Waterloo, Waterloo, ON, Canada; ^3^ University of Toronto, Toronto, ON, Canada; ^4^ KITE Research Institute, Toronto Rehabilitation Institute ‐ University Health Network, Toronto, ON, Canada; ^5^ Alzheimer Society of B.C., Vancouver, BC, Canada; ^6^ Vancouver Coastal Health Research Institute, Vancouver, BC, Canada; ^7^ Centre for Aging SMART, Vancouver Coastal Health Research Institute, Vancouver, BC, Canada; ^8^ University of British Columbia, Vancouver, BC, Canada; ^9^ Djavad Mowafaghian Centre for Brain Health, Vancouver, BC, Canada; ^10^ University of Saskatchewan, Saskatoon, SK, Canada; ^11^ McMaster University, Hamilton, ON, Canada; ^12^ Alzheimer Society of BC, Vancouver, BC, Canada

## Abstract

**Background:**

There is limited research focused on lifestyle interventions for people living with dementia and recent systematic reviews primarily focus on the impact of exercise on cognition. However, functional abilities and quality of life (QoL) are the outcomes most consistently prioritized by people living with dementia, care partners, and healthcare professionals. We conducted a systematic review to understand the impact of two lifestyle interventions (physical activity, nutrition) on the functional abilities, quality of life, and nutritional status (nutrition interventions only) of people living with dementia living in the community. The results presented here focus on the effects of nutrition interventions.

**Method:**

Systematic literature searches for peer‐reviewed intervention studies were conducted in four databases (MEDLINE, EMBASE, Scopus, CINAHL). Articles were exported to Covidence, where duplicates were removed and two independent reviewers performed study selection, data extraction, and risk of bias assessments. A narrative synthesis was conducted.

**Result:**

Six studies met the inclusion criteria. Five studies compared nutrition interventions to usual care. Interventions included education/counselling on nutrition through various delivery modes; one study also included coaching to reduce care partner stress and encourage protein supplementation. Sample sizes (*n* = 12 – 946) and follow‐up periods varied substantially across studies (3 weeks – 12 months). Five studies assessed nutritional status, all of which used the Mini‐Nutritional Assessment. The two largest studies, a cluster‐randomized trial with 946 participants (intervention = 448; control=498) and a non‐randomized controlled trial with 225 participants (intervention = 151; control=74) found significantly better nutritional status in the intervention compared to control groups. Results showed no change for two studies and data collection was incomplete for one study. Other outcomes measures included: QoL (*n* = 2 studies), activities of daily living (ADL)/instrumental ADL (*n* = 3 studies), falls (*n* = 1 study), grip strength (*n* = 1 study), timed up and go test (*n* = 1 study), lower body strength (*n* = 1 study), and step test (*n* = 1 study).

**Conclusion:**

Existing literature suggests that nutritional status can be improved through nutrition interventions, supporting a role for nutrition risk screening and intervention among people living with dementia. Additional large‐scale studies are needed to understand the impact of these interventions on nutritional status, QoL, and functional abilities while considering barriers to access.

